# Supplementary material for: The Roles of Standing Genetic Variation and Evolutionary History in Determining the Evolvability of Anti-Predator Strategies
Source: PLoS One. 2014 Jun 23;9(6):e100163. doi: 10.1371/journal.pone.0100163 (PMC4067307; doi:10.1371/journal.pone.0100163)
Supplement: Table S1 — Analysis of Variance (calculated using the anova() function in the R 3.0.2 base package) on a linear model*† testing the effects of SGV and EH and their interaction on Shannon's diversity among populations at the 200,000th update of Phase 2 evolution. (DOCX) [file pone.0100163.s007.docx]

| **Effect** | **DF** | **Mean squares** | **F-value** | **P-value** |
| --- | --- | --- | --- | --- |
| SGV | 2 | 1.45 | 2.45 | 0.09 |
| EH | 1 | 34.74 | 59.72 | < 0.0001 |
| SGV × EH | 2 | 2.65 | 2.28 | 0.11 |
| Error | 174 | 0.582 | -- | -- |

*Multiple R^2^ = 0.45

†Model: *$y\sim\beta_{0}+\beta_{1}SGV+\beta_{2}EH+\beta_{3}(SGV\times EH)+\varepsilon$*
