# Supplementary material for: The Roles of Standing Genetic Variation and Evolutionary History in Determining the Evolvability of Anti-Predator Strategies
Source: PLoS One. 2014 Jun 23;9(6):e100163. doi: 10.1371/journal.pone.0100163 (PMC4067307; doi:10.1371/journal.pone.0100163)
Supplement: Table S3 — Permutational multiple analysis of variance (PerMANOVA) output describing effects on SGV, EH, and PT (and all first-order interactions) on outcomes of ecological competitions (see Figure 2 ). 999 permutations were used. (DOCX) [file pone.0100163.s009.docx]

| **Fixed effect** | **DF** | **Mean squares** | **F-value** | **P-value** |
| --- | --- | --- | --- | --- |
| SGV | 2 | 0.203 | 1.54 | 0.228 |
| EH | 1 | 102.238 | 775.88 | < 0.0001 |
| PT | 1 | 0.026 | 0.20 | 0.668 |
| SGV × EH | 2 | 1.817 | 13.79 | < 0.0001 |
| SGV × PT | 2 | 0.370 | 2.81 | 0.062 |
| EH × PT | 1 | 3.313 | 25.14 | < 0.0001 |
| Residual | 1790 | 0.132 | -- | -- |

R^2^ = 0.32
