# Supplementary material for: The Roles of Standing Genetic Variation and Evolutionary History in Determining the Evolvability of Anti-Predator Strategies
Source: PLoS One. 2014 Jun 23;9(6):e100163. doi: 10.1371/journal.pone.0100163 (PMC4067307; doi:10.1371/journal.pone.0100163)
Supplement: Table S4 — AIC, LRT output, and model selection processes for linear mixed-effects models describing change in A. total prey instructions; proportion moves; C. proportion turns and D. proportion looks. Variance functions are shown with traits modeled (in italics). (DOCX) [file pone.0100163.s010.docx]

| **Compared** | **df** | | **AIC** | | | **LR** | **LRT P-val** | **Model pref.** |
| --- | --- | --- | --- | --- | --- | --- | --- | --- |
| *A. Total instructions:* $\sigma_{\beta2}^{2}$~ EH | | | | | | | | |
| Model 1 & Model 2 | 17 | 16 | 672.35 | | 678.09 | 7.74 | 0.0054 | 1, 1 chosen |
| *Proportion moves:* $\sigma_{\beta2}^{2}$~ EH | | | | | | | | |
| Model 1 & Model 2 | 17 | 15 | 238.75 | | 238.33 | 3.58 | 0.17 | none, 2 chosen |
| Model 2 & Model 3 | 15 | 13 | 238.33 | | 234.90 | 0.58 | 0.75 | none, 3 chosen |
| Model 3 & Model 4 | 13 | 12 | 234.90 | | 233.83 | 0.92 | 0.34 | none, 4 chosen |
| Model 4 & Model 5 | 12 | 10 | 233.83 | | 255.28 | 25.47 | < 0.0001 | 4, 4 chosen |
| *C. Proportion turns:*$\sigma_{\beta2}^{2}$~ EH*;*$\sigma_{\beta3}^{2}$~ SGV | | | | | | | | |
| Model 1 & Model 2 | 19 | 17 | 515.69 | | 514.63 | 2.95 | 0.23 | none, 2 chosen |
| Model 2 & Model 3 | 17 | 14 | 514.63 | | 510.37 | 1.74 | 0.63 | none, 3 chosen* |
| *D. Proportion looks:* $\sigma_{\beta2}^{2}$~ EH*;*$\sigma_{\beta3}^{2}$~ SGV | | | | | | | | |
| Model 1 & Model 2 | 19 | 17 | 568.97 | 565.89 | | 0.91 | 0.63 | none, 2 chosen |
| Model 2 & Model 3 | 17 | 14 | 565.89 | 562.62 | | 2.73 | 0.43 | none, 3 chosen |
| Model 3 & Model 5 | 14 | 12 | 562.62 | 565.69 | | 7.07 | 0.03 | 3, 3 chosen |

*All remaining first-order interactions significant, so no further model comparison.
