# Supplementary material for: The Roles of Standing Genetic Variation and Evolutionary History in Determining the Evolvability of Anti-Predator Strategies
Source: PLoS One. 2014 Jun 23;9(6):e100163. doi: 10.1371/journal.pone.0100163 (PMC4067307; doi:10.1371/journal.pone.0100163)
Supplement: Table S5 — Random intercepts and slopes (linear contrasts) between levels of PT with parametric bootstrap 95% confidence intervals for models describing change in A. total prey instructions; B. proportion moves; C. proportion turns; and D. proportion looks. Also included are marginal and conditional R2 and residual standard deviations with parametric bootstrap 95% CI. (DOCX) [file pone.0100163.s011.docx]

| **Trait** | **Coefficient** | **Std. dev.** | **Lower 95%** | **Upper 95%** |
| --- | --- | --- | --- | --- |
| *A. Total instructions:* $R_{m}^{2}$*= 0.66;* $R_{c}^{2}$*= 0.94* | | | | |
|  | slope | 0.36 | 0.32 | 0.39 |
|  | intercept | 0.40 | 0.36 | 0.44 |
|  | residuals | 0.21 | 0.19 | 0.22 |
| *B. Proportion moves:* $R_{m}^{2}$*= 0.58;* $R_{c}^{2}$*= 1.0* | | | | |
|  | slope | 0.03 | 0.03 | 0.03 |
|  | intercept | 0.59 | 0.53 | 0.66 |
|  | residuals | 0.13 | 0.12 | 0.14 |
| *C. Proportion turns*$R_{m}^{2}$*= 0.23;* $R_{c}^{2}$*= 0.97* | | | | |
|  | slope | 2.13e-9 | 1.90e-9 | 2.35e-9 |
|  | intercept | 0.66 | 0.59 | 0.73 |
|  | residuals | 0.23 | 0.21 | 0.24 |
| *D. Proportion looks:* $R_{m}^{2}$*= 0.11;* $R_{c}^{2}$*= 0.95* | | | | |
|  | slope | 0.20 | 0.18 | 0.22 |
|  | intercept | 0.94 | 0.85 | 0.1.04 |
|  | residuals | 0.10 | 0.09 | 0.10 |
