# Supplementary material for: The Roles of Standing Genetic Variation and Evolutionary History in Determining the Evolvability of Anti-Predator Strategies
Source: PLoS One. 2014 Jun 23;9(6):e100163. doi: 10.1371/journal.pone.0100163 (PMC4067307; doi:10.1371/journal.pone.0100163)
Supplement: Appendix S1 — S1.1: Genetic diversification during Phase 2 evolution. S1.2: Linear mixed effects models describing evolutionary change in traits. (DOCX) [file pone.0100163.s012.docx]

**Appendix (S1)**

***S1.1 Genetic diversification during Phase 2 evolution***

Over the 200,000 updates of Phase 2 evolution, *clone* SGV populations increased in diversity (Shannon’s Diversity Index), and *intermediate* and *high* SGV populations decreased in diversity, with all trends stabilizing near the 40,000^th^ update (Figure S1). Both standing genetic variation (SGV) and evolutionary history (EH) affected diversity values at the end of Phase 2 evolution. Diversity was similar across SGV treatments for *predator* EH populations, but was higher in *intermediate* and *high* than in *low* SGV populations for *no predator* EH populations. Shannon’s Diversity was higher over all for *no predator* than for *predator* EH populations (Figure S1; Table S1).

***S1.2 Linear mixed effects models describing evolutionary change in traits***

Linear mixed effects models describing change in prey instructions across Phase 2, compared using LRT with a parametric bootstrap. Models are nested, with Model 1 being the full model (all first and second-order interactions included), and Model 6 being the simplest (no interactions included). Models 2-5 include some (Models 3-5) or all (Model 2) first-order interactions. Interaction terms included were those determined to be statistically significant (parametric bootstrap confidence intervals around coefficients did not overlap zero). LRT outputs, AIC for each model, and model selection processes are given in Table S4, along with variance functions included in the chosen model for each trait.

Model 1 (full model):

$$y\sim\beta_{0ij}+\beta_{1ij}+\beta_{1}PT+\beta_{2}EH+\beta_{3}SGV+\beta_{4}(PT\times EH)+\beta_{5}(PT\times SGV)+$$

$$\beta_{6}(EH\times SGV)+\beta_{7}(PT\times EH\times SGV)+\varepsilon_{ijk},$$

*$\beta_{0ij}\sim(\beta_{0j},\sigma_{\beta_{0}}^{2}),\beta_{1ij}\sim(\beta_{1j},\sigma_{\beta_{1}}^{2})$*

Model 2:

$$y\sim\beta_{0ij}+\beta_{1ij}+\beta_{1}PT+\beta_{2}EH+\beta_{3}SGV+\beta_{4}(PT\times EH)+\beta_{5}(PT\times SGV)+$$

$$\beta_{6}(EH\times SGV)+\varepsilon_{ijk},$$

*$\beta_{0ij}\sim(\beta_{0j},\sigma_{\beta_{0}}^{2}),\beta_{1ij}\sim(\beta_{1j},\sigma_{\beta_{1}}^{2})$*

Model 3:

*$y\sim\beta_{0ij}+\beta_{1ij}+\beta_{1}PT+\beta_{2}EH+\beta_{3}SGV+\beta_{4}(EH\times SGV)+\varepsilon_{ijk},$*

*$\beta_{0ij}\sim(\beta_{0j},\sigma_{\beta_{0}}^{2}),\beta_{1ij}\sim(\beta_{1j},\sigma_{\beta_{1}}^{2})$*

Model 4:

$$y\sim\beta_{0ij}+\beta_{1ij}+\beta_{1}PT+\beta_{2}EH+\beta_{3}SGV+\beta_{4}(PT\times EH)+\varepsilon_{ijk},$$

*$\beta_{0ij}\sim(\beta_{0j},\sigma_{\beta_{0}}^{2}),\beta_{1ij}\sim(\beta_{1j},\sigma_{\beta_{1}}^{2})$*

Model 5 (fully reduced model):

*$y\sim\beta_{0ij}+\beta_{1ij}+\beta_{1}PT+\beta_{2}EH+\beta_{3}SGV+\varepsilon_{ijk},$*

*$\beta_{0ij}\sim(\beta_{0j},\sigma_{\beta_{0}}^{2}),\beta_{1ij}\sim(\beta_{1j},\sigma_{\beta_{1}}^{2})$*

*β_0ij_* are the randomly varying intercepts, and *β_1ij_* are the randomly varying slopes across PT levels.
